# Supplementary material for: What Competencies Does a Community Occupational Therapist Need in Neurorehabilitation? Qualitative Perspectives
Source: Int J Environ Res Public Health. 2022 May 17;19(10):6096. doi: 10.3390/ijerph19106096 (PMC9141779; doi:10.3390/ijerph19106096)
Supplement: Supplementary file 1 [file ijerph-19-06096-s001.zip › ijerph-1591966-Supplementary S1.pdf]

Interview guide

Greetings, thanks for acceptance, informed consent process (explaining study and interview goals).
Begin interview by asking some personal-professional information. Name, age, time in the profession, current work functions.

Dimension: Community-neurorehabilitation links

- When we think of community occupational therapy, how do you place yourself as an occupational therapist? What do you think about it?
- How would you describe your professional trajectory and your community links?

Dimension: Knowledge

- What theories or models do you habitually use?
- What knowledge should an occupational therapist have when working in neurorehabilitation?
- What theoretical background do you associate with community work?

Dimension: Knowledge

- What procedures do you habitually do?
- What type of evaluations do you use in your practice? How do you do them?
- Can you describe the interventions you typically do?
- How do you link your intervention with community space?
- What community actions do occupational therapists do in your work space?

Dimension: Attitudes

- What values predominate in OT practice in your area?
- What attitudes do you believe to be essential for your neurorehabilitation work?

Dimension: Elements to improve

- What key aspects should be improved to implement occupational therapy in neurorehabilitation?
